# Group social rank is associated with performance on a spatial learning task

**DOI:** 10.1098/rsos.171475

**Published:** 2018-02-14

**Authors:** Ellis J. G. Langley, Jayden O. van Horik, Mark A. Whiteside, Joah R. Madden

**Affiliations:** Centre for Research in Animal Behaviour, Psychology, University of Exeter, Exeter, UK

**Keywords:** cognition, individual differences, social rank, learning performance, pheasant, group

## Abstract

Dominant individuals differ from subordinates in their performances on cognitive tasks across a suite of taxa. Previous studies often only consider dyadic relationships, rather than the more ecologically relevant social hierarchies or networks, hence failing to account for how dyadic relationships may be adjusted within larger social groups. We used a novel statistical method: randomized Elo-ratings, to infer the social hierarchy of 18 male pheasants, *Phasianus colchicus*, while in a captive, mixed-sex group with a linear hierarchy. We assayed individual learning performance of these males on a binary spatial discrimination task to investigate whether inter-individual variation in performance is associated with group social rank. Task performance improved with increasing trial number and was positively related to social rank, with higher ranking males showing greater levels of success. Motivation to participate in the task was not related to social rank or task performance, thus indicating that these rank-related differences are not a consequence of differences in motivation to complete the task. Our results provide important information about how variation in cognitive performance relates to an individual's social rank within a group. Whether the social environment causes differences in learning performance or instead, inherent differences in learning ability predetermine rank remains to be tested.

## 1. Background

Characterizing variation in cognitive performances is pertinent to our understanding of the evolution of cognition [1,2]. An individual's cognitive performance may correspond to their social rank. Social rank arises from interactions between dominant and subordinate individuals in groups [3], and can influence an individual's access to resources [4], stress [5], and opportunities for learning [6,7], all of which may influence an individual's performance on a cognitive task.

Dominant individuals typically perform more efficiently on operant learning (starlings, *Sturnus vulgaris* [8]), spatial learning (mice [9,10]; meadow voles, *Microtus pennsylvanicus* [11]) and spatial memory tasks (mountain chickadees, *Poecile gambeli* [12]). However, such studies have relied on dyadic relationships between pairs of individuals, or concentrated on small groups, which may be simplistic and hence not reflect the network of relationships naturally observed in larger social groups [13]. Therefore, we are lacking an understanding of how variation in cognitive performance may be manifested in relation to real-world social dynamics.

When learning performance has been considered in the context of a group, results have been found that are contrary to the prediction that the more dominant individuals would be better learners. Subordinate individuals outperformed dominant individuals by making fewer errors on complex problem solving (long-tailed macaques, *Macaca fascicularis* [14]) and reversal learning tasks [15]. It is possible that this switch in the direction of the relationship is due to the difference in tasks deployed. For example, reversal learning and inhibitory control tasks can be used to assay cognitive/behavioural flexibility [16,17]. The ability to flexibly respond to changes when contingencies are altered is governed by separate neuronal pathways to acquisition learning [18]. Alternatively, the relationship between cognitive performance and social rank reported in the Bunnell *et al*.'s studies may have been complicated by the experimentally induced instabilities in social structure. Individuals were continually removed and re-introduced to the social group during the study period, therefore increasing social pressure for dominants who were attempting to maintain their rank [15], thus confounding the relationship between social rank and cognitive performance.

Our understanding of the relationship between social rank and cognitive performance can also be confused by the use of inappropriate methods to construct hierarchies, particularly those that don't consider whole groups of individuals or which rely on the outcomes of small numbers of interactions. This may explain why a number of studies failed to find a relationship between social rank and learning performance [19–21]. Critically, there are few guidelines for assessing the reliability of an inferred dominance hierarchy [22]. In order to understand how an individual's social rank relates to their cognitive performance, it is necessary to remove these confounds of variable test design and consider social status in more naturalistic multi-individual groupings.

The pheasant, *Phasianus colchicus*, provides a suitable system to explore the relationship between group social rank and variation in learning performance. In the wild, pheasants exhibit non-resource defence polygyny in which males compete for territories to attract females. Competition for territories takes the form of agonistic interactions and territorial display [23–25], and begins as early as December [26]. Females preferentially choose dominant males (see [27] for review), and non-harem holding males sexually harass females and achieve copulations through force [25,28]. Breeding season begins in March. Pheasants exhibit variation in spatial memory [29], although it is unclear whether this may be more important for territory holding males who know a specific area and its neighbouring areas in detail, or for satellite males who fail to command a territory and so utilize a much larger area more ephemerally. In captivity, males form stable dominance hierarchies for short periods (three months) but which are somewhat flexible, especially at the start of the breeding season [30]. Male dominance in captivity has been found to reflect the situation in the wild [31].

We tested adult male pheasants on a spatial discrimination task while they were members of a captive, multi-individual social hierarchy during the breeding season. We expect that measures of social rank are more meaningful during the breeding season while competition for resources is intense, compared with the winter months in which males aggregate [32] and measures of social rank may be more difficult to detect. We included the time it took individuals to begin the task as a measure of motivation to participate; a factor that may differ between individuals of different ranks. Males tend to occupy particular areas of the pen and so complete mixing does not always occur (E.J.G.L. 2015, personal observation). This may result in sparse interaction data, a problem when inferring social hierarchies [33,34]. To account for this we used a novel method of hierarchy inference which allowed us to determine the reliability of our inferred social hierarchy [22]. Previous studies compared the task performances of dyads (mice [9,10]; mountain chickadees [12]; meadow voles [11]) and show that the dominant individuals outperform the subordinate individuals. If we extend these findings, then we expect that spatial learning performance will be positively related to social rank in a complex, established and more realistic social environment.

## 2. Methods

### 2.1. Subjects and housing

In March 2015 at North Wyke Rothamsted Research Farm, Devon (50°77′ N, 3°9′ W), we caught wild adult pheasants and housed 18 males and 16 females in a large pen (20 m × 20 m) to give a sex ratio approximately mimicking that observed in the wild [35]. This pen contained refuges, branch shelters, perches and multiple feeders and drinkers. Individuals were identifiable by numbered patagial wing tags.

### 2.2. Cognitive testing

**Figure 1.**
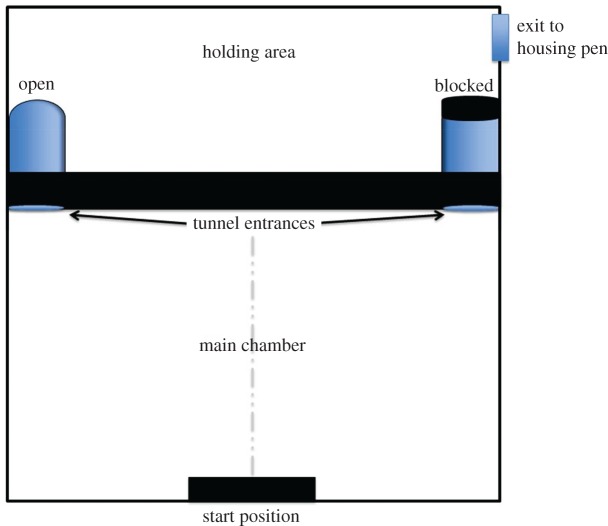
Aerial view of the left-right tunnel task testing arena.

From 9 to 13 April 2015, 15 males completed the ‘left-right tunnel’ cognitive task; one male did not engage with the task and two males were deliberately not tested because we expected their participation in a different experiment to influence their performance on this task. The task assayed discrimination learning in which individuals learned to associate a location (left or right) with the reward of leaving the testing arena (4 m × 4 m, figure 1). The testing arena was located within the housing pen but was in visual isolation from the regular housing. The testing arena comprised a main chamber which contained a pair of tunnels (arch shaped; H 20 cm, W 28 cm, L 60 cm), one on the left and right sides, 3.4 m apart, equidistant from a centre ‘starting point’. One tunnel was consistently blocked on the exit end (incorrect) while the other returned the individual to a holding area (correct), away from the experimenter and from which males could return to their regular housing at will. This chamber was novel to all individuals. During a trial, individual males were caught with a mesh net and placed on the starting point and oriented forwards. These procedures may have caused stress to the birds; however, upon placement on the starting point individuals did not exhibit behavioural indicators of stress such as panting or flight behaviour (inclusive of running). We recorded time to leave the starting point (t1), but due to lost data, only consider t1 from the first six trials as a measure of motivation to participate in the task. Males were unable to see the far end of either tunnel without lowering their head and individuals were considered to have made a choice when either: (1) they were within 1 m of the tunnel and lowered their head; or (2) when a part of their body entered the tunnel. The correct tunnel (left or right) was designated randomly for each individual. When individuals entered inside the incorrect tunnel they could return to the main testing chamber by their own volition and a trial was finished when the male exited through the correct tunnel. Therefore, males experienced exiting through the correct tunnel on every trial. For analyses, we only considered an individual's initial choice. Each individual received 14 trials in total. The first seven trials were conducted within one day; individuals were randomly selected for the first trial and then this order of testing was maintained for the remaining trials. The second seven trials were carried out four days later, following the same protocol as above. Therefore, inter-trial intervals were consistent between individuals.

### 2.3. Dominance interactions

From 20 to 28 May, we observed 367 agonistic interactions between males (table 1) via ad libitum sampling conducted by four visually concealed observers. This method of sampling is suitable to capture event behaviours, such as aggressive interactions [36]. Interactions had a clear winner and loser. Each observer focused their efforts on one quarter of the pen and communicated via two-way radio when interactions occurred across these quarters to ensure observations were not duplicated. During the first day of observations we watched the birds for 2.5 h and on the remaining seven days, we conducted two observation sessions of 30 min each (a.m. and p.m.). To generate the social ranks we used an extension of the Elo-rating method. In the original Elo-rating method, individuals begin with the same start rating and this is updated after each agonistic interaction [34,37]. The value each rating is updated by is dependent on the outcome of the interaction (won or lost) and the probability of that outcome occurring, relevant to both individuals' current Elo-rating. From these ratings individuals are organized into a hierarchy, allowing one to conduct parametric statistics if necessary because individuals' social ranks are associated with a continuous variable. The extension of this method; the randomized Elo-rating method [22], then allows one to assess whether an adequate number of interactions were recorded to infer a social hierarchy and quantify uncertainty in the inferred hierarchy from the generation of randomized interaction data. Due to the subjects participating in other separate experiments, there was a gap of approximately five weeks between cognitive testing and recording of dominance observations, but based on previous work [30] we expect that the social hierarchy remained relatively stable within the breeding season. We believe that by conducting observations while the group size was constant (and birds were not removed for brief periods because of cognitive testing) and there was no interference from researchers, interactions would be more representative of natural dominance relationships.

**Table 1.** Ethogram of agonistic interactions between male pheasants.

*agonistic*	
* chase*	aggressor (winner) runs towards opponent and opponent flees (loser)
* threat\lunge*	aggressor (winner) steps forwards and makes a sharp movement towards opponent, opponent flees or avoids (loser). Similar to the start of a *chase* but aggressor does not continue to run
* contact*	aggressor (winner) pecks opponent (loser) with the bill, usually directed at the head or neck, or aggressor (winner) jumps at opponent feet first to direct spurs at opponent (loser)
*submissive*	
* avoid*	an individual (loser) rapidly changes trajectory while walking and is within 3 m of another individual (winner) that is not showing any apparent signs of aggression

### 2.4. Statistical analysis

All analyses were conducted using R v. 3.1.1 [38]. Using the *aniDom* package [39] we generated ‘randomized Elo-ratings’ and assessed hierarchy uncertainty using the two methods described in Sanchez-Tojar *et al.* [22]: we firstly estimated repeatability of the individual Elo-ratings generated from replicated datasets (*n* = 1000) using the *rptR* package [40], with high repeatability scores indicating a steep hierarchy (high probability that a dominant individual wins a contest); secondly, we split the interaction dataset into two halves, computed 1000 individual ranks for each half using the randomized Elo-rating method and calculated the Spearman's Rank Correlation *r*_S_ between the ratings generated by the two halves. We report the mean *r*_S_ and 95% confidence interval range of the correlation values. These results indicated low levels of uncertainty in the data, therefore we used the mean of the randomized Elo-ratings from the full dataset in subsequent analyses, hereby referred to as ‘mean Elo-rating’. We used the *rptR* package [40] to assess whether males were repeatable in the time they took to engage in the task (t1). If individuals are consistent in the time taken to begin the task over multiple presentations, we can conclude that this assay is a meaningful measure of their motivation. Individuals exhibited a significant level of repeatability (*R* = 0.231 ± 0.104, *p* = 0.005)*.* Therefore mean t1, which was log-transformed to normalize the distribution, was used in subsequent analyses. The inclusion of mean t1 rather than t1 per trial also reduced the complexity of the subsequent model, which was necessary given the small sample size. We fitted a generalized linear mixed model (GLMM) with a binomial error structure and a logit link function using the *lme4* package [41] to assess whether social rank could explain learning performance with ‘Correct’ (1 yes/0 no) as the response variable, and trial, mean Elo-rating, first trial performance (correct: 1 yes/0 no) and mean time taken to begin the task (log mean t1), as explanatory variables. This model was fitted on 13 trials, after performance on the first trial was removed from the Correct variable and included as a separate explanatory variable. An interaction term between mean Elo-rating and trial was included to assess whether individuals of different social rank differ in their rate of learning. We included the first trial performance as an explanatory variable because the outcome of this trial was prior to the opportunity for learning but may affect subsequent performance on the task. The inclusion of mean t1 (log) controlled for motivation to participate in the task. The model failed to converge, this was resolved by standardizing mean Elo-rating and trial by converting them to *z* scores [42]. Trial was nested within individual as a random effect to control for repeated choices of individuals, and to allow the explanatory variables to vary randomly between individuals (random intercepts and random slopes model). The minimum adequate model was reached by comparing models based on log likelihood using backward stepwise deletion of non-significant variables. Results of the full model are provided at http://doi.org/10.24378/exe.21. We calculated odds ratios (OR) from the exponential of *b*_1_ and deduced confidence intervals (CIs) for variables in the minimum adequate model. To visualize results we plotted curves predicted from binary logistic regression models for each third of the hierarchy. To ensure that we were capturing variation in cognitive performance rather than other factors, we attempted to fit subsequent GLMMs using a binomial error structure and logit link function. Firstly, we fitted a model to check whether individuals of varying rank differed in their motivation to participate, with Correct (1 yes/0 no) as the response variable and an interaction term between mean Elo-rating with time to begin the task (log mean t1) as explanatory variables. Second, we fitted a model to check there were no rank-related biases; individuals of higher rank were more likely than lower ranking individuals to choose correctly on the first trial, with Correct as the response variable and an interaction term between mean Elo-rating and first trial performance (correct: 1 yes/0 no). The models, however, failed to converge. Therefore, we conducted a Spearman's rank correlation between mean Elo-rating and mean t1 (log); and used binary logistic regression models, fitted with a binomial error structure and logit link function with Correct (1 yes/0 no) as the response variable and trial as an explanatory variable to generate learning curve coefficients. The model outputs are provided at http://doi.org/10.24378/exe.21. From the coefficients of each model we calculated the predicted probability that individuals would choose correctly on the first trial (*X* = 1) using the formula 1/(1 + EXP(−(*b*_0_ + *b*_1_))). We conducted a Spearman's rank correlation between mean Elo-rating and *X* = 1.

## 3. Results

### 3.1. Social hierarchy

Our observations were sufficient to produce a steep, reliable hierarchy. The repeatability score of our randomized Elo-ratings was 0.978 and the mean correlation coefficient obtained by splitting the interaction data was 0.751 (95% CI: 0.554, 0.909).

### 3.2. Spatial learning performance

**Figure 2.**
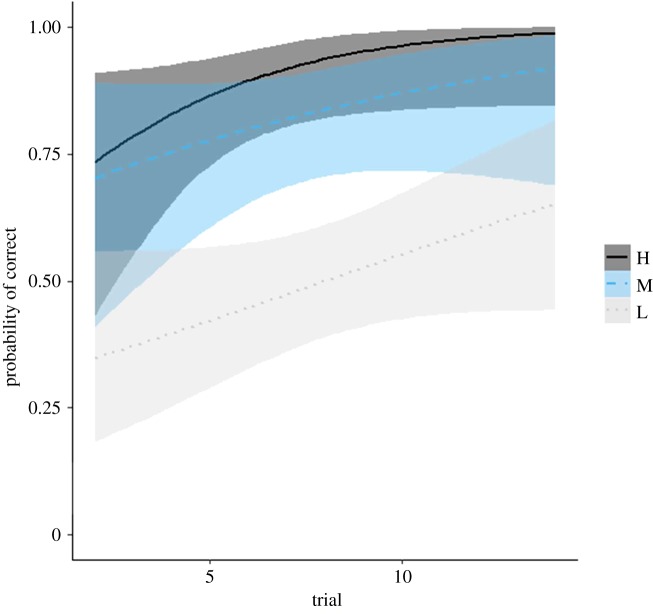
Predicted probability of choosing correctly on a spatial discrimination task with increasing trial number for male pheasants. Curves were drawn using a binary logistic regression model; for the three males that achieved a mean Elo-rating in the upper third (H); the five males that achieved an Elo-rating within the middle third (M); and the seven males that achieved an Elo-rating within the lower third (L), of the mean Elo-rating range. Mean Elo-ratings were deduced from 1000 randomized Elo-ratings. The shaded areas indicate 95% confidence intervals for each curve.

The percentage of correct choices per individual on the 13 trials ranged from 21 to 100% correct. The interaction between social rank and trial was not significant (table 2 and figure 2), indicating that individuals of different social rank did not learn the spatial discrimination task at different rates. However, social rank and trial number were significant main effects in the model (table 2 and figure 2). Specifically, higher ranking males were more likely to choose correctly and the probability of choosing correctly increased with trial number, indicative of learning. Performance on the first trial was a significant main effect in the model (GLMM: first trial, Wald *χ*^2^ = 4.956, *p* = 0.026); males that chose correctly on their first trial were more likely to choose correctly on subsequent trials (table 2). Motivation to engage in the task did not affect the probability that a male would choose correctly (GLMM: mean t1 (log), Wald *χ*^2^ = 1.815, *p* = 0.178).

**Table 2.** Minimum adequate model from generalized linear mixed model on the effects of performance on first trial (correct: 1 yes/0 no), social rank (mean Elo-rating *z*-score) and trial (*z*-score) on success on a spatial discrimination task by male pheasants tested individually but while housed in a social group, with odds ratios (OR), lower (Lo CI) and higher confidence intervals (Hi CI). Individual (variance = 1.353) and trial (variance = 0.128) were included as random effects to allow explanatory variables to vary randomly between individuals (random slopes model).

	estimate	s.e.	Wald *χ*^2^	*p*	OR	Lo CI	Hi CI
explanatory variable							
intercept	0.939	0.364					
first trial	1.372	0.580	4.956	0.026	3.942	0.128	2.616
trial	0.637	0.241	7.391	0.007	1.891	0.120	1.154
mean Elo-rating	1.023	0.309	9.282	0.002	2.781	0.360	1.686

The motivation to engage in the cognitive task, deduced from mean t1, was not significantly related to social rank (Spearman's rank correlation: mean Elo-rating with mean t1 (log), *r*_S_ = −0.042, *n* = 15, *p* = 0.887). The predicted probability of an individual choosing correctly on the first trial was not related to social rank (Spearman's rank correlation: mean Elo-rating and *X* = 1, *r*_S_ = 0.176, *n* = 15, *p* = 0.531).

## 4. Discussion

The higher a male pheasant's social rank, the better their performance on a spatial discrimination task. Social rank was not related to the rate at which males chose correctly, but males' performances improved with experience, suggesting that the task captured capacities for spatial learning. Motivation to engage in the task was not related to social rank, nor did it relate to learning performance. There was no relationship between social rank and performance on the first trial, but individuals that chose correctly on their first trial had better performance overall. This suggests that in pheasants, the ability to learn to discriminate between spatial locations corresponds positively to an individual's social rank while in a group, rather than to differences in motivation or rank-related biases for a location. By considering the social hierarchy of a large group, these findings provide us with a broader view on how cognitive performances correspond to complex social systems, in which individuals have multiple relationships.

Our results complement findings from other species in which high levels of aggression and competitive ability exhibited by an individual have been positively linked to their learning and memory performance [8–12]. Contrary to these studies which concentrated on dyads [9,10,12] or small groups [8], we considered a range of rank positions in a social hierarchy, providing more information. We may envisage that consistently winning or consistently losing contests effects cognitive performance positively and negatively, respectively. There is very little information, however, on how the intermediate ranks, which are those individuals that experience both winning and losing contests, may vary in their cognitive performance. Furthermore, studies of pairs or small groups neglect important social effects such as the role of bystanders on outcomes of social interactions [13]. Therefore, these social ranks and their associated cognitive performances may not be fully representative of how this relationship manifests in natural situations. It is possible that the social hierarchy we inferred from the dominance observations had changed since the cognitive testing was conducted. However, previous work on pheasants shows that when group composition is held constant, hierarchies become well established [24,43], and although Mateos *et al*. do not comment explicitly on the duration of hierarchy stability, other Galliformes demonstrate stable hierarchies when housed over similar periods to our study (up to 20 weeks in domestic chickens, *Gallus gallus domesticus* [44]; at least three weeks in jungle fowl, *Gallus gallus* [45]).

In contrast to our findings, Bunnell *et al.* report the opposite relationship between rank and cognitive performance, in which lower ranking macaques, *Macaca fascicularis*, were more proficient on reversal learning tasks [15]. This finding, however, may be due to the unnatural and frequent changes made to the macaques' group composition, the added stressor of which may have had a more adverse effect on higher ranking individuals' cognitive performance than that of lower ranking individuals. Alternatively, the reversal test faced by the macaques may better indicate cognitive flexibility or inhibitory control; abilities important for lower ranking individuals as they regularly experience negative repercussions from those of higher rank [46]. It would be interesting to investigate if reversal learning is negatively related to social rank within our group system.

The positive relationship between performances on the cognitive task with social rank in our pheasants may be a consequence of our testing paradigm and not a result of differences between social ranks in cognitive ability. Individuals may have differed in their motivation to leave the testing chamber (e.g. whether they have preferential access to females). We assume that higher ranking males may have had less contested access to females in the communal pen, as females have been found to prefer dominant males [28]. Although this is not something that we quantified, it is possible that higher ranking males habitually guarded females in the communal pen, causing them to be more motivated to choose the correct tunnel. Additionally, low ranking males may have been less motivated to return to the communal pen where they could be subject to aggression. However, the probability of choosing the correct tunnel generally increased with each trial for males of all social ranks so we suggest that males valued this reward equally.

Higher ranking individuals may have been more likely to choose correctly on their first trial, just by chance, and thus had an advantage for the remainder of the task. However, the predicted probability of making a correct choice on the first trial was not related to social rank, suggesting that males of different rank were equally likely to choose correctly on the first trial. Furthermore, after choosing incorrectly on the first trial, individuals experienced the correct tunnel as they exited the main testing chamber. Therefore, we can rule out that individuals of higher rank had an advantage on this task.

Alternatively, low ranking males may have had poorer memory for the correct tunnel or were slower to learn its location because of the stress associated with living as a subordinate in a hierarchy [5,47] which impedes cognitive performances [48]. It is likely that the lower ranking pheasants were in receipt of a higher level of aggression from conspecifics than higher ranking males [23] and consequently, their performance on the task was impaired. Future research could explore whether pheasants differ in stress levels according to their social rank and if this mechanism explains variation in learning performance.

In an ecologically relevant, multi-individual mixed-sex social environment, we found that, for male pheasants, variation in accuracy but not rate of individual learning performance on a spatial discrimination task was correlated positively with their social rank. Perhaps male pheasants that were inherently good at learning about space become dominant because they are better able to recall spatial features and so more efficiently establish and hold a territory. Alternatively, males may establish their dominance position independently of their performance in spatial memory tasks, but once they attain a dominant position, they may express better spatial learning because they have had more opportunity to learn spatial cues in a reliable and consistent territory (i.e. they are able to learn to learn). Whether social rank drives differences in cognitive performance or instead inherent differences in cognitive performance predetermine rank remains to be explored.

## Data accessibility

Datasheets supporting this manuscript are available at the Open Research Exeter repository: http://doi.org/10.24378/exe.21.
